# The next frontier of athletic intelligence in sports medicine and exercise science

**DOI:** 10.17159/2078-516X/2026/v38i1a27263

**Published:** 2026-10-15

**Authors:** H Noorbhai

**Affiliations:** Physical Activity, Sport, and Recreation (PhASRec), Faculty of Health Sciences, North-West University, Potchefstroom, South Africa

**Keywords:** AI, markerless motion capture, athlete monitoring, biomechanics, decision support, ethics

## Abstract

Artificial intelligence (AI) is rapidly transforming sports medicine and exercise science, offering unprecedented capabilities in markerless motion capture, automated exercise prescription, diagnostic imaging and integrated athlete monitoring. These advances have the potential to democratise biomechanical analysis, scale clinical expertise to underserved populations and shift injury management from reactive treatment to predictive prevention. However, the pace of adoption carries significant risks, including technological dependency without methodological literacy, the entrenchment of global inequities through biased training datasets and models, the erosion of clinician-athlete relationships and a widening governance vacuum over data ownership and algorithmic accountability. This commentary argues that the next frontier of athletic intelligence will be defined not by technological sophistication alone, but by the wisdom, inclusivity and ethical resolve with which the sports medicine community deploys it. Disciplined engagement (rather than uncritical adoption or reflexive avoidance) is the potential path forward.

The convergence of artificial intelligence (AI), sensor technology and cloud computing is reshaping the landscape of sports medicine and exercise science at a pace that few disciplines have experienced before. Where practitioners once relied on subjective observation, stopwatches and periodic laboratory testing, the field now has access to continuous physiological streams, markerless motion capture and large language models (LLMs) capable of synthesising clinical and biomechanical data in real time. This is not a distant promise but a present reality, unevenly distributed and incompletely understood.

The purpose of this commentary is to issue a dual provocation: first, that the sports medicine community stands at a generational opportunity to redefine athletic intelligence; and second, that the window for responsible adoption is closing faster than most of us realise. The ‘carrot’ is extraordinary, but the ‘stick’ is unforgiving.

## The carrot: what AI makes possible

Consider the state of biomechanical assessment a decade ago. Three-dimensional motion analysis required a dedicated laboratory, retroreflective markers and hours of post-processing. Today, deep learning-based pose estimation systems can extract kinematic data from standard video footage with sufficient accuracy for clinical screening and coaching feedback.^[1,2]^ Markerless motion capture has evolved from a novelty to a validated methodology, with recent work demonstrating moderate-to-high reliability across lower-limb joints during athletic tasks.^[2]^ The democratisation of biomechanics is no longer theoretical, as it is being deployed on smartphones, on the sidelines and on playing fields across the world. In South Africa and across much of sub-Saharan Africa, where three-dimensional (3D) laboratories are concentrated in a handful of urban academic centres, smartphone-based markerless capture offers a tangible route to biomechanical screening in school sport, community clubs and public-sector physiotherapy and rehabilitative clinics that have historically had no access to such tools.^[1]^

Equally transformative is the emergence of generative AI in exercise prescription. A systematic review now confirms that LLMs can produce personalised training recommendations with reasonable accuracy, although questions around reliability, contextual adaptation and clinical safety remain open.^[3]^ The implication is significant: AI does not replace the exercise scientist or sports physician, but it compresses the time between assessment and intervention and scales expertise to populations that have historically been underserved (which is critical in LMIC (Low- and Middle-Income Countries) contexts).

In diagnostics, AI-enhanced imaging is redefining the sensitivity of injury detection. Algorithms trained on musculoskeletal MRI datasets can detect subtle structural changes that the human eye misses, while AI-driven electrocardiogram analysis is improving the accuracy of pre-participation cardiac screening among athletes.^[4]^ These are not incremental gains but a categorical shift in what is detectable, and therefore, preventable.

Perhaps the most compelling frontier lies in integrating multiple data streams into unified athlete intelligence platforms. Wearable sensors that generate continuous physiological, biomechanical, and neuromuscular data can be combined with environmental, psychological, and tactical information to create holistic decision-support systems for practitioners.^[5]^ The athlete of tomorrow will be understood through a living, dynamic (personalised) model of their performance, health and readiness; updated in real time and interpreted through AI. A concrete example is the consolidation of wearable-derived training load, heart rate variability, sleep, and neuromuscular fatigue data into a single decision-support dashboard, which can flag an elevated risk of anterior cruciate ligament injury, muscle strain, or stress fracture before symptoms appear, shifting the clinical task from treating an injury to pre-empting it.^[5]^

## The stick: what do we risk?

For all its promise, the AI revolution in sports medicine carries risks that must be confronted with equal candour.

The first risk is technological dependency without methodological literacy. As AI tools become more accessible, there is a growing temptation among practitioners to treat algorithmic outputs as ground truth. A markerless motion capture system may produce plausible joint angles, but without an understanding of its error margins, training data biases and environmental constraints, the clinician risks acting on noise rather than signal.^[6]^ The ease of data collection must never be confused with the quality of data interpretation.

The second risk is inequity. The AI tools currently reshaping sports medicine are overwhelmingly developed in, and for, high-resource sporting environments in North America, Europe and parts of East Asia. African sports medicine, South American community sport and developing-world exercise science are largely absent from the training datasets, validation studies and product roadmaps that define these technologies. If the field does not act deliberately to ensure inclusive development, AI will entrench existing disparities rather than resolve them. This is a structural concern.

The third risk concerns the erosion of the clinician-athlete relationship. AI excels at pattern recognition, prediction and classification. It does not understand context, trust, fear, motivation or the lived experience of injury and recovery. The multidisciplinary sports medicine team functions through human judgement, communication and relational intelligence.^[6]^ If AI is positioned as a replacement for these capacities rather than an augmentation of them, we will make faster, lower-quality decisions, and athletes will be the ones who pay the price.

Figure 1 renders this argument as a single organising schema. Flowing from AI into sports medicine and exercise science, it sets four opportunities: democratised biomechanics (markerless motion capture), scaled exercise prescription, enhanced diagnostics and integrated athlete-intelligence platforms; against four parallel risks: technological dependency, global inequity, relational erosion and a governance vacuum. Athletic intelligence sits at the centre, and the schema resolves downward into disciplined engagement, operationalised through three practices [critical evaluation (methodological literacy), inclusive development (validation in LMIC contexts), and ethical governance (consent, transparency, and accountability)]. These are bound together by a continuous cycle of interrogation and validation.

Finally, the field faces a governance vacuum. There is no consensus framework for the ethical deployment of AI in sports medicine. Questions of data ownership, algorithmic transparency, informed consent for biometric monitoring and liability for AI-assisted clinical errors remain largely unanswered. The speed of technological adoption is outpacing the development of regulatory and ethical guardrails. As such, the longer this gap persists, the harder it will be to close (Figure 1). Progress here does not require inventing governance from first principles. Existing health-AI frameworks can be adapted to sport by vesting ownership of biometric data with the athlete, mandating algorithmic transparency and explainability, requiring explicit informed consent for continuous biometric monitoring, as well as defining clear lines of accountability for AI-assisted clinical decisions.

## A way forward

The path forward is neither wholesale embrace nor cautious avoidance but disciplined engagement. Sports medicine and exercise science must cultivate a new form of professional competency: the ability to critically evaluate, responsibly deploy and continuously interrogate AI-driven tools within clinical and coaching practice. This competency does not currently exist in most university curricula, and its absence is felt most acutely among early-career practitioners who are adopting these tools the fastest.^[6]^ Concretely, this means embedding structured AI-literacy modules (covering model validation, error and bias, data governance and critical appraisal) into postgraduate sports medicine and exercise science curricula, rather than leaving practitioners to acquire these competencies informally.

Journals in the fields of sports medicine and exercise science play a pivotal role (and some are already rising to the challenge). Yet the field needs a more concerted effort to solicit and publish rigorous validation studies of AI technologies in diverse sporting and clinical contexts, with particular attention to LMIC settings where the need is greatest, and the evidence is thinnest. They must also hold space for critical commentary that challenges techno-optimism without retreating into technophobia. One implementable step is for journals to adopt a minimum reporting standard for AI validation studies; for example, requiring authors to report training-data demographics, external and local validation, error margins and code or data availability, drawing on established checklists such as TRIPOD-AI and CLAIM. For LMIC settings specifically, progress can be accelerated through low-cost smartphone-based tools, cultural integration of open-source science, federated data initiatives that enable sites to contribute to model training without surrendering sensitive data, and capacity-building partnerships that pair well-resourced laboratories with local clinicians and researchers.

## Conclusion

Instead of the next frontier of athletic intelligence being defined by the sophistication of the technology alone, it will be defined by the wisdom with which we use it, the honesty with which we confront its limitations and the resolve with which we insist that it serves all athletes (and not only those who can afford it). Ultimately, this approach moves from a technological challenge towards a moral one.

## Figures and Tables

**Fig. 1:**
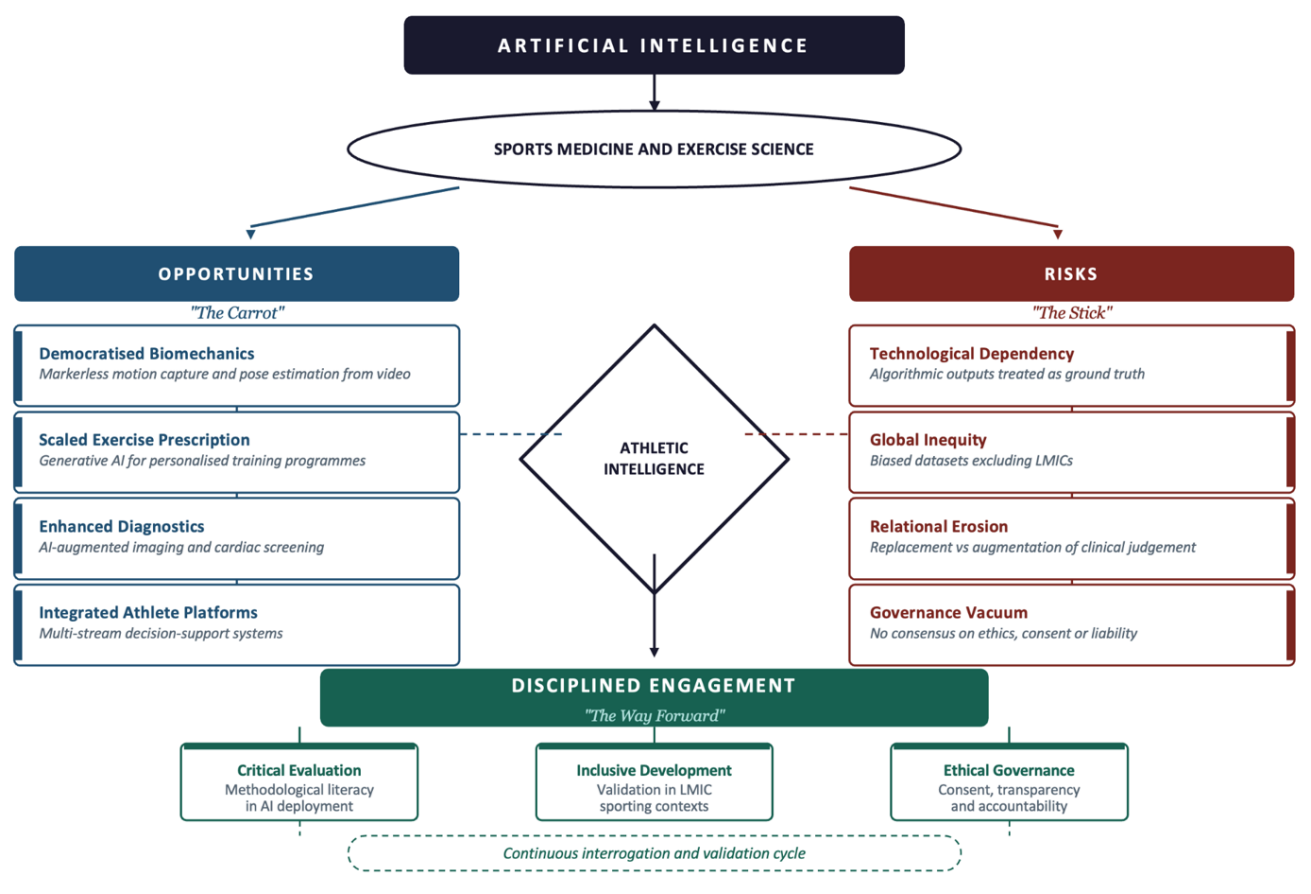
A conceptual framework for the next frontier of athletic intelligence in sports medicine and exercise science
